# Preventive effects of chronic exogenous growth hormone levels on diet-induced hepatic steatosis in rats

**DOI:** 10.1186/1476-511X-9-78

**Published:** 2010-07-26

**Authors:** Ying Qin, Ya-ping Tian

**Affiliations:** 1 Department of Clinical Biochemistry, Chinese People's Liberation Army General Hospital, 28 Fu-Xing Road, Beijing, China

## Abstract

**Background:**

Non-alcoholic fatty liver disease (NAFLD), which is characterized by hepatic steatosis, can be reversed by early treatment. Several case reports have indicated that the administration of recombinant growth hormone (GH) could improve fatty liver in GH-deficient patients. Here, we investigated whether chronic exogenous GH levels could improve hepatic steatosis induced by a high-fat diet in rats, and explored the underlying mechanisms.

**Results:**

High-fat diet-fed rats developed abdominal obesity, fatty liver and insulin resistance. Chronic exogenous GH improved fatty liver, by reversing dyslipidaemia, fat accumulation and insulin resistance. Exogenous GH also reduced serum tumour necrosis factor-alpha (TNF-alpha) levels, and ameliorated hepatic lipid peroxidation and oxidative stress. Hepatic fat deposition was also reduced by exogenous GH levels, as was the expression of adipocyte-derived adipokines (adiponectin, leptin and resistin), which might improve lipid metabolism and hepatic steatosis. Exogenous GH seems to improve fatty liver by reducing fat weight, improving insulin sensitivity and correcting oxidative stress, which may be achieved through phosphorylation or dephosphorylation of a group of signal transducers and activators of hepatic signal transduction pathways.

**Conclusions:**

Chronic exogenous GH has positive effects on fatty liver and may be a potential clinical application in the prevention or reversal of fatty liver. However, chronic secretion of exogenous GH, even at a low level, may increase serum glucose and insulin levels in rats fed a standard diet, and thus increase the risk of insulin resistance.

## Background

Non-alcoholic fatty liver disease (NAFLD) is a metabolic disorder characterized by fatty infiltration of the liver in the absence of alcohol consumption. It is the most common cause of chronic liver disease and represents a spectrum of liver diseases, which include simple fatty liver, steatohepatitis, and cirrhosis [1]. NAFLD is present in 10-24% of the world's population and its prevalence is even greater in obese individuals, ranging from 57.5 to 74.0%.

Hepatic steatosis is a hallmark of NAFLD and is caused by the accumulation of lipids, particularly triglycerides, in the liver. Hepatic steatosis, usually considered as an early stage of NAFLD, is generally benign, relatively non-aggressive and reversible. The symptoms are not obvious and the disease is often overlooked. However, because hepatic steatosis can progress to fibrosis (in 20-40% of patients), cirrhosis (in 30% of patients) or hepatocellular carcinoma [2-5], early prevention and treatment are essential. Regulation of excessive lipid synthesis and uptake is thought to be an effective intervention for NAFLD. Thus, lipid-lowering agents are promising pharmacological therapies for hepatic steatosis [6].

Growth hormone (GH) has a pronounced lipolytic effect, particularly in abdominal fat [7]. Previous studies have shown that inhibition of endogenous GH signalling might perturb lipid metabolism and induce liver steatosis [8]. The low physiological level of GH is closely linked to steatosis in NAFLD patients [9]. Several case reports have shown that administration of recombinant GH can improve fatty liver in GH-deficient (GHD) patients by normalizing serum triglyceride and cholesterol levels [10,11]. Moreover, pharmacological doses of recombinant GH can overcome hepatic GH resistance in patients with chronic liver disease, increase serum insulin-like growth factor (IGF)-1, and improve the protein catabolic state [12]. However, chronic GH administration may cause a sustained deterioration of glucose metabolism as a consequence of the lipolytic effect of GH, resulting in enhanced oxidation of lipid substrates and increased insulin resistance (IR) [12].

Adipocyte-derived adipokines such as adiponectin, leptin and resistin, are essential regulators of inflammation and the progression of fibrosis in various chronic liver diseases, and may be used in the treatment of NAFLD [13-16]. Specifically, adiponectin could alleviate obesity-induced hepatomegaly and steatosis (fatty liver) [13]. Leptin promoted hepatic fibrogenesis through upregulation of transforming growth factor-beta (TGF-beta) in the liver [14,15]. Hepatic resistin is involved in the development of obesity and IR, and accelerates hepatic inflammation and fibrosis [16]. GH is an important modulator in the production of adipocyte-derived adipokines and can indirectly improve NAFLD by regulating adipokine production [13,17-19]. GH replacement therapy may be more effective than administration of individual adipokines in the prevention and treatment of NAFLD.

Here, we investigated whether early GH administration has the preventive effects on hepatic steatosis (an early stage of NAFLD) in rats, and explored the underlying mechanisms. We also discuss limitations of GH administration for hepatic steatosis. Viral vectors can induce longer-lasting effects than administration of recombinant protein and avoid the inconvenience of repetitive subcutaneous injections. Therefore, we used GH gene delivery technology rather than injection of recombinant GH. The GH1 gene (a human GH [hGH] gene; GenBank accession number NM_000515) coding sequence (cds) was transferred *in vivo *by recombinant adeno-associated viral vectors pseudotyped with viral capsids from serotype 1 (rAAV2/1).

## Results

### Food intake, body composition, liver wet weight and hepatic index (HI)

**Figure 1:**
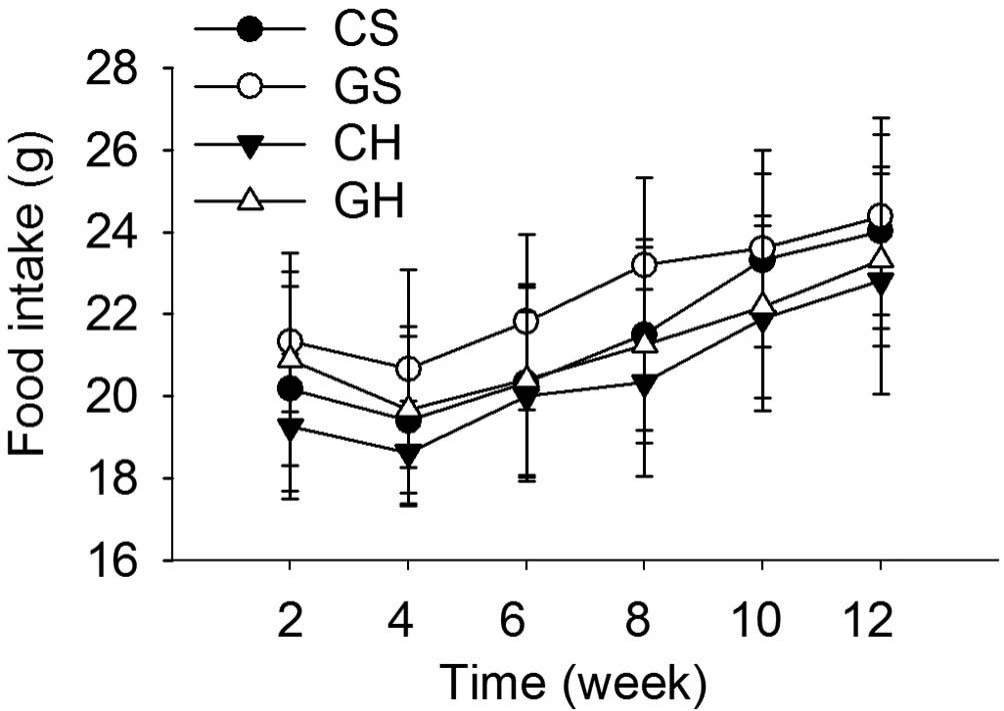
**Daily food intake**. Error bars represent standard deviations. (CS group, n = 6; GS group, n = 6; CH group, n = 10; GH group, n = 10).

The daily food intake was virtually identical among the four groups of rats (Fig. 1); however, the high-fat diet caused significant body weight gain compared with the control diet and the body weight of the CH (fat liver) group was significantly greater than that of the CS (control) group at week 12 (p < 0.01) (Table 1). Injection of rAAV2/1-CMV-GH1 resulted in a small but not significant increase in body weight in the GS group compared with the CS group (Table 1). In contrast, in the high-fat diet fed rats, exogenous GH levels reduced the body weight gain compared with the control group (GH vs. CH, p < 0.05) (Table 1), even though food intake was marginally increased in the GH group.

**Table 1:** Body composition and HI in each group of rats

	CS	GS	CH	GH
**TWB (g)**	433.07 ± 22.00^b^	443.93 ± 23.51^a^	474.81 ± 26.37^†^	451.12 ± 22.73^a^
**BMI (g/cm^2^)**	1.31 ± 0.15^a^	1.23 ± 0.11^b^	1.49 ± 0.16*	1.34 ± 0.13^a^
**LWW (g)**	11.3 ± 1.55^c^	12.48 ± 1.57^b^	16.04 ± 1.95^‡^	13.43 ± 1.74*^b^
**HI**	2.60 ± 0.26^c^	2.83 ± 0.44^b^	3.40 ± 0.55^‡^	2.97 ± 0.31^†b^
**VF (g)**	6.50 ± 0.71^c^	5.00 ± 0.67^†c^	9.51 ± 0.63^‡^	8.08 ± 0.98^†c^
**VF (%)**	1.51 ± 0.19^c^	1.13 ± 0.19^†c^	2.01 ± 0.18^‡^	1.80 ± 0.27*^a^

TBW: total body weight; BMI: body mass index; LWW: liver wet weight; HI: Hepatic index; VF: visceral fat; VF (%): percentage of visceral fat. Error bars represent standard deviations. *p < 0.05, ^†^p < 0.01 and ^‡^p < 0.001 *vs*. the CS group. ^a^p < 0.05, ^b^p < 0.01 and ^c^p < 0.001 *vs*. the CH group. (CS group, n = 6; GS group, n = 6; CH group, n = 10; GH group, n = 10).

As expected, the high-fat diet increased visceral fat weight and visceral fat percentage in the CH group, compared with the CS group (both, p < 0.001). Meanwhile, chronic exogenous GH improved the body composition and decreased visceral fat (VF) weight and VF percentage (VF%) in the GH group compared with the CH group (p < 0.001 and p < 0.05, respectively). Hepatomegaly, which is common in NAFLD, is determined by liver wet weight (LWW) and the HI. LWW and HI were significantly increased in the CH group than in the CS group (both, p < 0.001). Chronic exogenous GH levels prevented hepatomegaly, because LWW and HI decreased in the GH group compared with the CH group (both, p < 0.01) (Table 1). However, HI was higher in the GS group than in the CS group, although not significantly (Table 1).

### Serum GH and Insulin-like growth factor 1 (IGF-1)

**Figure 2:**
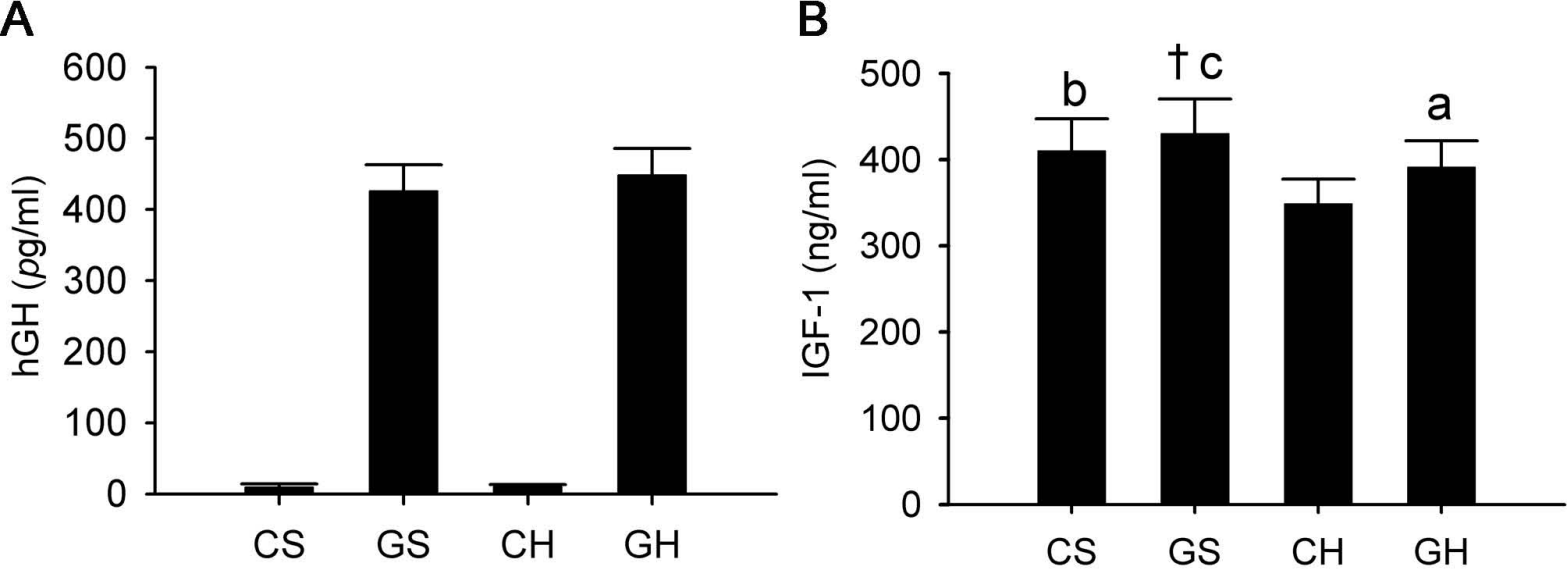
**Effects of rAAV2/1-CMV-GH1 and high-fat feeding on serum hormone levels**. hGH: human growth human; IGF-1: insulin-like growth factor 1. Serum levels of hGH (A) and IGF-1 (B). *p < 0.05, ^†^p < 0.01 and ^‡^p < 0.001 *vs*. the CS group. ^a^p < 0.05, ^b^p < 0.01 and ^c^p < 0.001 *vs*. the CH group. (CS group, n = 6; GS group, n = 6; CH group, n = 10; GH group, n = 10).

GH1 gene expression can be sustained for at least 6 months after the injection of rAAV2/1-CMV-GH1, as we have already reported [20]. Serum hGH was detected in the GS and GH groups. As a result, serum IGF-1 levels were higher in the GS and GH groups than in the CS and CH groups. Interestingly, the high-fat diet decreased the serum IGF-1 levels in the CH group, which was reversed by the injection of rAAV2/1-CMV-GH1 in the GH group compared with the CH group (Fig. 2).

### Hepatic and serum lipid and lipoprotein parameters

The high-fat diet significantly increased the serum levels of total cholesterol (TC) (p < 0.001), triglyceride (TG) (p < 0.001) and low-density lipoprotein cholesterol (LDL-C) (p < 0.001) and decreased the serum level of high-density lipoprotein cholesterol (HDL-C) in the CH group compared with the CS group. Chronic exogenous GH markedly reduced the serum TC (p < 0.01), TG (p < 0.001) and LDL-C levels (p < 0.01) and increased, albeit not significantly, the HDL-C levels (p > 0.05) in the GH group compared with the CH group, indicating that exogenous GH levels reversed the dyslipidaemia induced by the high-fat diet (Table 2). Similar improvements in lipid profiles were also noted in the GS group compared with the CS group. However, chronic exogenous GH also increased the serum glucose (p < 0.05) and insulin levels, although not significantly, in the GS group relative to the CS groups, and may increase the risk of IR, which was reflected by changes, although not significant, in the homeostasis model assessment of IR (HOMA-IR).

**Table 2:** Serum biochemical parameters

	CS	GS	CH	GH
ALT (U/l)	31.53 ± 7.43^c^	29.62 ± 4.45^c^	78.65 ± 20.41^‡^	42.78 ± 11.73^c^
AST (U/l)	70.35 ± 8.54^c^	66.40 ± 7.80^c^	128.01 ± 24.97^‡^	84.54 ± 11.76^c^
TG (mmol/l)	2.77 ± 0.14^c^	2.15 ± 0.37*^c^	4.02 ± 0.55^‡ ^	3.18 ± 0.35^c^
TC (mmol/l)	2.03 ± 0.21^c^	2.05 ± 0.22^c^	2.69 ± 0.34^‡^	2.24 ± 0.19^b^
HDL-C (mmol/l)	1.20 ± 0.26^b^	1.36 ± 0.30^c^	0.79 ± 0.25^†^	0.92 ± 0.14*
LDL-C (mmol/l)	0.27 ± 0.22^c^	0.26 ± 0.24^c^	1.10 ± 0.47^‡^	0.69 ± 0.12*^b^
Glucose (mmol/l)	4.25 ± 0.86^c^	4.65 ± 0.85*^b^	6.01 ± 0.90^‡^	5.28 ± 0.57*^a^
Insulin (μU/ml)	36.28 ± 4.10^c^	38.55 ± 3.78^b^	45.18 ± 3.94^‡^	40.00 ± 3.14^b^
HOMA-IR	6.93 ± 1.52^c^	8.00 ± 1.17^c^	12.09 ± 2.19^‡^	9.26 ± 1.32*^b^

ALT: alanine aminotransferase; AST: aspartate aminotransferase; TG: triglyceride; TC: total cholesterol; HDL-C: high-density lipoprotein cholesterol; LDL-C: low-density lipoprotein cholesterol; HOMA-IR: the homeostasis model assessment of insulin resistance. Error bars represent standard deviations. *p < 0.05, ^†^p < 0.01 and ^‡^p < 0.001 *vs*. the CS group. ^a^p < 0.05, ^b^p < 0.01 and ^c^p < 0.001 *vs*. the CH group. (CS group, n = 6; GS group, n = 6; CH group, n = 10; GH group, n = 10).

### Histological evaluation

**Figure 3:**
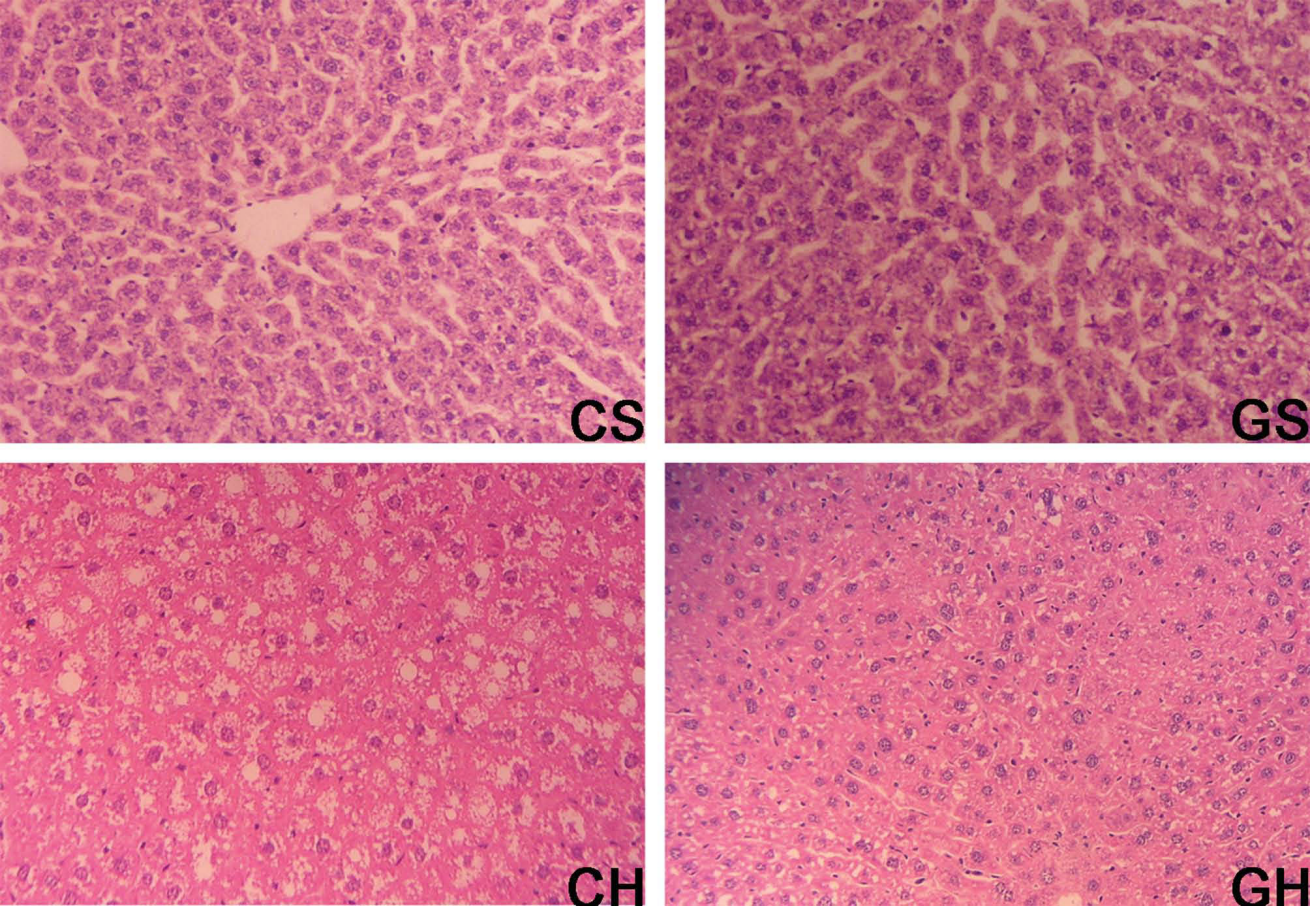
**Effects of rAAV2/1-CMV-GH1 and high-fat feeding on liver histology**. Lipid infiltration or significant hepatocytes abnormalities were not observed in the CS or GS groups. Numerous vacuoles were present in hepatocytes in the CH group. In contrast, the GH group contained fewer and smaller vacuoles compared with the CH group. (haematoxylin/eosin staining; magnification ×40).

Photomicrographs of hepatic specimens stained with H&E are shown in Figure 3. Lipid accumulation was not observed in the CS group (Fig. 3A). The injection of rAAV2/1-CMV-GH1 viral particles did not cause obvious hepatic changes in the GS group (Fig. 3B). As would be expected, the high-fat diet induced hepatic lipid accumulation in the CH group (Fig. 3C), with a mean grade of 2 (Table 3). The livers from the CH group also showed microvesicular or macrovesicular steatosis around the periportal zone, necrosis, and inflammation, along with enlarged hepatocytes (Fig. 3C). Fat deposition in this group was classified as mixed. Similar degenerative changes were noted in the GH group (Fig. 3D), but to a much lesser extent; the grade of lipid accumulation was 1, which was significantly lower than that in the CH group (p < 0.05). Fat deposition in the GH group was classified as microvesicular. Inter-observer agreement was 0.86.

**Table 3:** Grading of steatosis.

Group	**No**.	Steatosis grades			
		
		0	1	2	3
CS	6	6 (6)	0 (0)	0 (0)	0 (0)
GS	6	6 (6)	0 (0)	0 (0)	0 (0)
CH	10	0 (0)	3 (4)	6 (5)	1 (1)
GH	10	2 (0)	5 (7)	3 (3)	0 (0)

Kappa value = 0.86, Se (*_k_*) = 0.075, p < 0.001

### Tumour necrosis factor-alpha (TNF-alpha), lipid peroxidation (MDA) and oxidative stress

Serum TNF-alpha levels were remarkably elevated in the CH group compared with the CS group (p < 0.001). Although exogenous GH levels did not affect the serum TNF-alpha level in the GS group, it did partly prevent the high-fat diet-induced increase in serum TNF-alpha level in the GH group, as the serum TNF-alpha level was significantly lower in the GH group than in the CH (p < 0.001) (Table 4). The hepatic MDA level was also significantly higher in the CH group compared with the GH group (p < 0.001), as well as the CS and GS groups (Table 3). The hepatic levels of superoxide dismutase (SOD) (p < 0.01), glutathione peroxidase (GSH) (p < 0.001) and catalase (CAT) (p < 0.001) were significantly greater in the GH group compared with the CH group. However, hepatic nitric oxide synthase (NOS) (p < 0.001) was significantly lower in the GH group than in the CH group. Therefore, chronic exogenous GH significantly decreased the effects of stress oxidative in the liver (Table 4).

**Table 4:** TNF-alpha and markers of oxidative stress.

	CS	GS	CH	GH
TNF-alpha (pg/ml)	13.33 ± 2.08^c^	14.43 ± 2.45^c^	27.58 ± 3.88^‡^	17.63 ± 3.94*^c^
MDA (μM)	3.15 ± 0.82^c^	3.68 ± 1.09^c^	7.98 ± 1.56^‡^	4.01 ± 1.05^c^
SOD (U/ml)	2.63 ± 1.61^a^	3.88 ± 2.07^b^	0.85 ± 0.50*	3.48 ± 2.15^b^
NOS (μM)	1.85 ± 0.63^c^	0.78 ± 0.59^c^	8.72 ± 1.63^‡^	2.33 ± 0.83^c^
CAT (nmol/min/ml)	3082.77 ± 662.41^c^	4360.43 ± 843.47^‡c^	419.27 ± 191.40^‡c^	2409.18 ± 483.67*^c^
GSH (nmol/min/ml)	443.47 ± 43.71^c^	494.08 ± 54.81*^c^	8.95 ± 2.99^‡^	435.29 ± 46.72^c^

TNF-alpha: peroxisome proliferator-activated receptor-alpha; MDA: malondialdehyde; SOD: superoxide dismutase; NOS: nitric oxide synthase; CAT: catalase; GSH: glutathione peroxidase. Error bars represent standard deviations. *p < 0.05, ^†^p < 0.01 and ^‡^p < 0.001 *vs*. the CS group. ^a^p < 0.05, ^b^p < 0.01 and ^c^p < 0.001 *vs*. the CH group. (CS group, n = 6; GS group, n = 6; CH group, n = 10; GH group, n = 10).

### Bacterial translocation and systemic infection

Bacterial translocation from mesenteric lymph nodes (MLNs) is considered one of the main events in the pathogenesis of spontaneous bacterial peritonitis and other infections in cirrhosis [21]. TNF-alpha is known to be involved in bacterial translocation in rats with cirrhosis. Therefore, we tested whether bacterial translocation acted as a stimulus for TNF-alpha production in our model. In fact, the MLN cultures were negative in all four experimental groups, which indicates that bacterial translocation was not responsible for the elevated levels of TNF-alpha.

### Effects of chronic exogenous GH levels on the expression of ob-rb, resistin and adipoR2 *in vivo*

**Figure 4:**
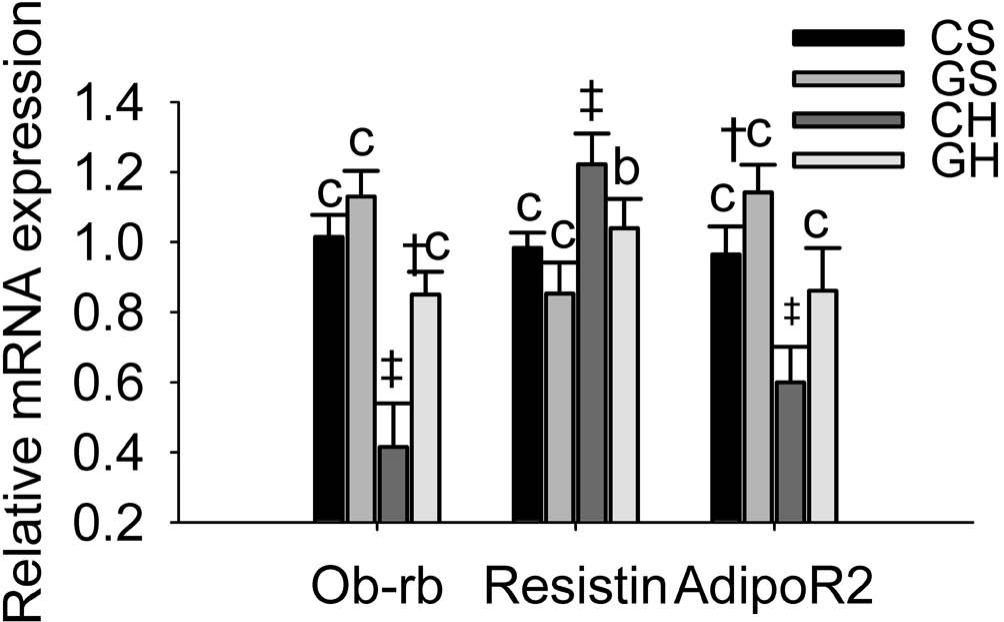
**Effects of rAAV2/1-CMV-GH1 and high-fat feeding on mRNA expression of hepatic ob-rb, resistin and adipoR2**. Ob-rb: the full-length leptin receptor isoform; AdipoR2: adiponectin receptor 2. Error bars represent standard deviations. *p < 0.05, ^†^p < 0.01 and ^‡^p < 0.001 *vs*. the CS group. ^a^p < 0.05, ^b^p < 0.01 and ^c^p < 0.001 *vs*. the CH group. (CS group, n = 6; GS group, n = 6; CH group, n = 10; GH group, n = 10).

The full-length leptin receptor isoform, ob-rb, contains intracellular motifs required for the activation of the JAK/STAT signal transduction pathway, and is considered to be the functional receptor [22]. The effects of adiponectin are principally mediated through the R1 and R2 adiponectin receptors (adipoR1 and adipoR2), and adipoR2 is predominantly found in the liver [23]. Figure 4 shows the differential expression of ob-rb, resistin, and adipoR2 mRNA among the four groups of rats. The high-fat diet significantly decreased hepatic ob-rb (p < 0.001) and adipoR2 (p < 0.001) mRNA expression and increased resistin mRNA expression (p < 0.001). Meanwhile, exogenous GH levels significantly increased hepatic ob-rb and adipoR2 mRNA expression (both, p < 0.001) and decreased hepatic resistin mRNA expression (p < 0.01) in the GH group compared with the CH group (Fig. 4).

### Effects of chronic exogenous GH levels on the expression of hepatic signal transducers and activators

**Figure 5:**
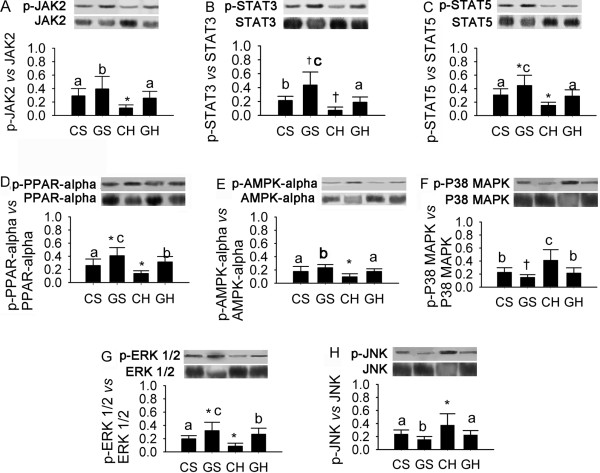
**Effects of rAAV2/1-CMV-GH1 and high-fat feeding on hepatic expression of p-JAK2, p-STAT3, p-STAT5, p-PPAR-alpha, p-AMPK-alpha, p-P38 MAPK, p-ERK1/2 and p-JNK**. p-JAK2: phospho-Janus kinase 2; JAK2: total Janus kinase 2; p-STAT3: phospho-signal transducer and activator of transcription 3; STAT3: total signal transducer and activator of transcription 3; p-STAT5: phospho-signal transducer and activator of transcription 5; STAT5: total signal transducer and activator of Transcription 5; p-PPAR-alpha: phospho-peroxisome proliferator-activated receptor-alpha; PPAR-alpha: total peroxisome proliferator-activated receptor-alpha; p-AMPK-alpha: phospho-adenosine monophosphate-activated protein kinase-alpha; AMPK-alpha: total adenosine monophosphate-activated protein kinase-alpha; p-P38 MAPK: phospho-p38 mitogen-activated protein kinase; P38 MAPK: total p38 mitogen-activated protein kinase. p-ERK1/2: phospho-extracellular signal-regulated kinase 1/2; ERK1/2: total extracellular signal-regulated kinase 1/2; p-JNK: phospho-c-Jun NH2-terminal kinase; JNK: total c-Jun NH2-terminal kinase. *p < 0.05, ^†^p < 0.01 and ^‡^p < 0.001 *vs*. the CS group. ^a^p < 0.05, ^b^p < 0.01 and ^c^p < 0.001 *vs*. the CH group. (CS group, n = 6; GS group, n = 6; CH group, n = 10; GH group, n = 10).

The high-fat diet induced marked changes in the expression of a group of signal transducers and activators in the CH group compared with the CS group, which were reversed by exogenous GH. Figure 5 shows the changes in expression of hepatic signal transducers and activators in the four different groups. Western blotting revealed that the significant inductions in the p-JAK2 (p < 0.01), p-STAT3 (p < 0.001), p-STAT5 (p < 0.01), p-AMPK-alpha (p < 0.001), p-ERK2/1 (p < 0.001) and p-PPAR-alpha (p < 0.01) relative to the expressions of the corresponding total proteins in the GH group compared with that in the CH group. Furthermore, the relative protein expression of p-P38 MAPK (p < 0.001) and p-JNK (p < 0.001) relative to those of P38 MAPK and JNK was significantly lower in the GH group than in the CH group (Fig. 5). Collectively, these results indicate that changes in the expression of signal transducers and activators induced by the high-fat diet could be reversed by exogenous GH levels.

## Discussion

IR plays a crucial role in NAFLD [24], which is closely associated with obesity and is often accompanied by marked abdominal adiposity [25]. Fat accumulation in the liver and IR cause and potentiate each other, creating a vicious cycle of metabolic dysfunction, resulting in the development and progression of NAFLD [26]. Meanwhile, reducing TG accumulation and improving insulin sensitivity can offer an effective therapeutic strategy for NAFLD [26].

GH is an anabolic hormone with pronounced lipolytic effects, particularly in abdominal fat [7]. Previous studies have shown that GH can reduce *de novo *lipogenesis and improve lipid profiles by decreasing TG and TC concentrations, and increasing HDL-C concentrations [27]. GH inhibits the activity of lipoprotein lipase in adipose tissue [28] and increases fat oxidation [29]. GHD may also cause hepatic steatosis and non-alcoholic steatohepatitis [10,11]. It was also reported that GH and IGF-1 are both closely associated with NAFLD [9]. Low hepatic GH levels may lead to the development of hepatic steatosis in patients with NAFLD, whereas low serum IGF-1 levels contribute to fibrosis [9]. It has been reported that GH administration can decrease abdominal visceral fat and trunk fat, increase lean body mass and reduce triglyceride levels, and thus improve NAFLD in GHD patients [10,11].

Here we examined whether chronic exogenous GH levels could improve hepatic steatosis in rats and we explored the underlying mechanism. NAFLD was characterized by hepatocyte lipid accumulation, which was visible under light microscopy as small vacuoles within the cytoplasm. The important features of NAFLD noted in the hyperlipidemic rats, including biochemical changes and classical histological lesions, were consistent with those in humans with NAFLD. Chronic exogenous GH levels were achieved in rats by the injection of rAAV2/1-CMV-GH1, as we have described previously [20]. As would be expected, serum IGF-1 was also elevated by rAAV2/1-CMV-GH1 injection.

Because excessive caloric intake plays an important role in the pathogenesis of NAFLD, food intake and body weight gain are the two primary targets of non-pharmacological interventions for NAFLD [26]. Our results showed that daily food intake was virtually identical in the CH and CS groups of rats, which eliminated the possible effects of hyperphagia on weight gain. The injection of rAAV2/1-CMV-GH1 decreased the abdominal fat weight in the GH group compared with the CH group, which suggests that exogenous GH could improve the body composition in rats fed a high-fat diet. The serum levels of LDL-C, TG and TC were decreased while HDL-C was increased in GH1-treated rats compared with the control rats. Although the injection of rAAV2/1-CMV-GH1 increased the serum levels of glucose and insulin in rats fed a standard diet (i.e., GS group), these increases were prevented in the rats fed the high-fat diet (i.e., GH group). Thus, although chronic exogenous GH may reduce insulin sensitivity in rats fed a standard diet, it seems to reverse IR in an animal model of fatty liver induced by a high-fat diet. The reduction in abdominal fat weight and prevention of IR might be involved in the mechanisms by which chronic exogenous GH protects against NAFLD induced by a high-fat diet.

The pro-inflammatory cytokine TNF-alpha plays important roles in the development of IR, and influences lipid metabolism [30]. As a result, TNF-alpha has emerged as a crucial factor underscoring liver damage in NAFLD. TNF-alpha inhibits the propagation of insulin receptor-initiated signals in hepatocytes and is known to promote IR and steatohepatitis in ob/ob mice and NAFLD patients [31,32]. TNF-alpha not only mediates the early stages of fatty liver disease, but also the transition to advanced stages of liver damage [33]. We found that chronic exogenous GH lowered the serum level of TNF-alpha, improved IR and lessened steatohepatitis in NAFLD rats.

Oxidative stress is considered to play an important role in the progression of simple steatosis to advanced liver damage, and is accompanied by degenerative changes in the enzymatic antioxidant defence systems [21,34]. Elevated lipid peroxidation leads to increased MDA generation in NAFLD [21]. In turn, MDA can stimulate the production of cytokines that participate in the activation of spindle cells and fibrogenesis [21,35]. At the same time, the low levels of SOD, GSH and CAT in animal models and patients with NAFLD suggest their increased utilization because of enhanced oxidative stress. We found that the high-fat diet increased the serum MDA level, and decreased the levels of hepatic antioxidants such as SOD, GSH and CAT, but increased NOS in the liver. Injection of rAAV2/1-CMV-GH1 reversed the changes in antioxidant activities induced by the high-fat diet and ultimately improved oxidative stress; which constitute another mechanism by which chronic exogenous GH levels prevents fatty liver.

The elevated levels of aminotransferases are the result of leakage from damaged cells, and are used as markers of liver injury, particularly for NAFLD [36]. Abnormal aminotransferase activity was noted in rats fed the high-fat diet, and was reversed by rAAV2/1-CMV-GH1 injection in the GH group, although GH1 gene therapy did not induce obvious changes in rats fed the standard diet. These results provide further evidence that chronic exogenous GH levels could improve hepatic steatosis in rats without obvious hepatotoxicity.

Hepatic GH signalling is essential to regulate intrahepatic lipid metabolism [8]. Activation of the GH receptor (GHR) on the target cells promote the association of JAK2 with the GHR, which initiates tyrosine phosphorylation of GHR and JAK2, and activates multiple signalling cascades by stimulating the phosphorylation of downstream signalling molecules that regulate the transcription of GH-responsive genes [37,38]. GH responsive processes include cellular transport, enzymatic activity and gene expression, which ultimately culminate in changes in hepatic lipid metabolism.

We found that chronic exogenous GH affected the phosphorylation of several hepatic signalling molecules, including JAK2/STAT3 (STAT5), p38-MAPK, AMPK, ERK1/2 and PPAR-alpha, signalling molecules that are closely involved in the pathogenesis of NAFLD [26,39-42]. For example, activation of the JAK2/STAT3 signalling pathway can reduce hepatic fat deposition and fibrosis [39], while inhibition of JNK and activation of the ERK1/2 MAPK pathways can improve steatohepatitis in rats [40]. The reduced phosphorylation of p38 MAPK is associated with improvements in hyperglycaemia and hyperinsulinaemia in the diabetic ob/ob mice after treatment with an antisense oligonucleotide against protein tyrosine phosphatase 1B (PTP1B) that plays a crucial role in the effect of glutathione depletion on lipid metabolism [41,42]. Meanwhile, the activation of AMPK can inhibit lipid synthesis, reduce hepatic TG accumulation, promote glucose metabolism and inhibit gluconeogenesis in the liver. Thus, the inhibition of AMPK phosphorylation may induce NAFLD [25]. PPAR-alpha regulates fatty acid-beta-oxidation and plays an important role in modulating hepatic TG accumulation [43]. Therefore, PPAR-alpha agonists have hepatoprotective effects and activation of PPAR-alpha signalling can reverse hepatic steatosis and fibrosis [6,44]. However, the PPAR-alpha agonists such as fibric acid derivatives (fibrates) treatment require almost life long consumption of the medication. The main side-effects of fibrates are gastrointestinal and muscular that cannot be neglected [45,46]. Our finding showed that the activation of PPAR-alpha by exogenous GH may also ameliorate NAFLD in rats without obvious side-effects, which suggested potential application value of GH in the prevention or reversal of fatty liver.

GH can also regulate the expression of adipocyte-derived hormones [13,17-19] by upregulating or downregulating the expression of these genes in NAFLD rats. These adipokines have been proposed as being associated with the inflammation and progression of fibrosis seen in NAFLD [13-16]. For example, GH regulates the expression of adiponectin and its receptors in adipocytes via the JAK2 and p38 MAPK pathways [13]. In fact, GH replacement therapy seems to be more suitable than adiponectin administration for the prevention and treatment of NAFLD because adiponectin activity is limited, despite the relatively high plasma concentrations of adiponectin (5-30 μg/ml). In the present study, exogenous GH upregulated hepatic ob-rb and adiponectin receptor 2 (adipoR2) mRNA expression, but significantly downregulated resistin mRNA expression in rats fed a high-fat diet compared with those fed a control diet. The changes in expression of these genes may further modulate hepatic lipid metabolism in rats fed a high fat diet and in turn prevent or reverse fatty liver.

There are several limitations to our study. For example, the molecular mechanisms underlying the effects of exogenous GH on NAFLD are complex and gene expression may be confounded by the duration and dosage of the rAAV2/1-CMV-GH1 treatment. Meanwhile, chronic exogenous GH may elevate serum glucose and insulin levels in vivo, and thus increase the risk of IR. In addition, the AAV vector has only been tested in human clinical trials, and its safety and efficiency need further investigation. Although this study offers a good starting point for the development of GH gene therapy for early prevention and treatment of NAFLD, many more studies are needed to confirm the applicability of the findings before the approach can be applied clinically.

## Conclusions

Chronic exogenous GH has preventive effects against hepatic steatosis and fatty liver, and may be realized through reduced fat weight, enhanced insulin sensitivity and correction of oxidative stress. These effects of GH may be achieved by its regulation of genes through the phosphorylation or dephosphorylation of a group of signal transducers and activators in several hepatic signal transduction pathways. However, exogenous GH may also increase serum glucose and insulin levels in rats, and thus increase the risk of insulin resistance.

## Methods

### Construction and production of the rAAV2/1 vector containing GH1

We used a previously described method to construct the rAAV2/1 vector containing GH1 [20,23,45,46]. Briefly, GH1 was cloned from a PCR product using 5'-CA**GAATTC**GCCACCATGGCTACAGGCTCCCGG-3' (forward primer) and 5'-CTGC**GTCGAC**GAAGCCACAGCTGCCCTC-3' (reverse primer) (*Eco*RI and Sa*l*I restriction sites are indicated in bold) from the template of a pUC19 plasmid DNA containing GH1 (Xinxiang Medical University, Henan Province, P. R. China). The GH1 DNA fragment (677 bp, including the 651-bp cds) was digested with Sa*l*Iand *Eco*RI and inserted into the Sa*l*I and *Eco*RI sites of the pSNAV2.0 vector (AGTCGene Technology Co. Ltd., P.R. China). rAAV2/1 production and purification were performed as previously described [47]. The viral genome particle titre (1.0 × 10^12 ^v.g./ml) was determined by a quantitative DNA dot blot method [48].

### Animals

Animal experiments were performed in accordance with the guidelines of the National Institutes of Health (Bethesda, MD, USA) and the Chinese People's Liberation Army General Hospital for the humane treatment of laboratory animals. All efforts were made to minimize the number of animals used and their suffering.

Thirty-two adult male Sprague-Dawley rats (180 ± 10 g) were obtained from the Institute of Laboratory Animal Sciences, Chinese Academy of Medical Sciences (CAMS) & Peking Union Medical College (PUMC) (Beijing, China). Rats were housed at 23°C with a 12-h light/dark cycle, and were allowed free access to food and water. Half of the rats were randomly selected and intravenously injected with a single dose of 1.25 × 10^11 ^rAAV2/1-CMV-GH1 viral particles into the tail vein. The remaining (control) rats were injected with a single dose of empty rAAV2/1 vectors. Two weeks later, the GH1 gene-treated rats were divided into two groups and fed either a standard (control) diet (4.5 g/100 g fat) (GS group, n = 6) or a high-fat diet to induce fatty liver (GH group, n = 10) consisting of 10% hog fat, 2.5% glucose, 2% cholesterol and 0.25% cholic acid added to normal chow for 10 weeks, as previously described [6]. Similarly, the control rats were also divided into two groups and fed the standard (control) diet (CS group, n = 6) or the high-fat diet to induce fatty liver (CH group, n = 10). Food intake and body weight were recorded during the experimental period.

At week 12 (i.e., 2 weeks after injection followed by 10 weeks of feeding), the rats were fasted overnight and sacrificed by anaesthesia with 10% chloral hydrate solution. Body length (distance from the nose to the anus [N-A distance]) and total body weight (TBW) were measured by an electronic balance (Scout Pro Balance; Ohaus, Pine Brook, NJ, USA) that was calibrated every day and an electronic digital calliper (Control Co., Friendswood, TX, USA). Body mass index (BMI) was calculated using the formula BMI = TBW/(BL)^2^. The perirenal and epididymal fat pads were pooled (visceral fat, VF) and weighed using a precision electronic balance (AV264; Ohaus). The VF weights of each rat were normalized to TBW by calculating the percentage as follows: weight/TBW × 100%. Fresh liver tissues were immediately dissected and some samples were prepared for histological analyses, while others were weighed and snap-frozen between blocks of dry ice and stored at -80°C. The HI was calculated as liver weight/TBW × 100%.

Blood was collected via the aorta and allowed to clot at room temperature for 60 min to form serum. The serum samples were then centrifuged at 3000 × *g *for 15 min at 4°C, and stored at -80°C until used for biochemical and hormone assays.

### Histological analyses

The freshly dissected rat livers were immediately fixed in 4% paraformaldehyde, dehydrated, embedded in paraffin, and sectioned. Formalin-fixed, paraffin-embedded sections were cut (5 μm thick) and mounted on glass slides. The sections were deparaffinized in xylene, stained with haematoxylin and eosin (H&E) using standard techniques, and examined by an investigator blind to the treatment group. Biopsies were classified into four grades based on fat accumulation using the classification method devised by Brunt et al [49], where grade 0 indicates no fat present in the liver, while grades 1 (light), 2 (mild) and 3 (severe) were defined as the presence of fat vacuoles in < 33%, 33-66% or >66% of hepatocytes, respectively. The pattern of fat deposition was classified as macrovesicular, microvesicular or mixed. Two experienced pathologists blinded to the experimental conditions evaluated all samples. Agreement between both pathologists was determined.

### Serum hormone and biochemical analysis

Serum human GH (hGH; Roche, Pleasanton, California, USA), TNF-alpha (R&D Systems, Minneapolis, MN, USA), IGF-1 (ADL, Alexandria, VA, USA) and insulin (ADL) was measured using enzyme-linked immunosorbent assay (ELISA) kits. All assay kits included quality controls. Each sample was assayed in duplicate. The serum levels of glucose, alanine aminotransferase (ALT), aspartate aminotransferase (AST), TG, TC, and HDL-C were measured using standard methods. LDL-C level was calculated using Friedwald's formula [50]. IR was assessed using HOMA-IR as blood glucose × blood insulin/22.5 [51,52].

### Lipid peroxidation and oxidative stress

To measure the level of hepatic MDA, 25 mg of liver tissue was added to 250 μl of radioimmunoprecipitation assay (RIPA) buffer containing protease inhibitors. This mixture was sonicated for 15 s at 40 V over ice and centrifuged at 1600 × *g *for 10 min at 4°C. The supernatant was used for analysis. MDA was quantified using the thiobarbituric acid reaction as described by Ohkawa [21,53], and measured using a thiobarbituric acid reactive substances (TBARS) assay (Cayman Chemical Co. Inc., MI, USA). The hepatic levels of SOD, CAT, GSH and NOS as oxidant/antioxidant biochemical parameters were also quantified using appropriate assays (Cayman Chemical Co. Inc.).

### Determination of bacterial translocation

Samples of MLNs and portal and peripheral blood were collected under sterile conditions before the rats were killed. The MLNs were homogenized in physiological (0.9%) saline, and 0.1 ml aliquots of the homogenate were cultured in MacConkey agar (Oxoid, Basingstoke, UK), Columbia sheep blood (Oxoid), and Esculin-Bile-Azide agar (Merck, Darmstadt, Germany), and incubated at 37°C for 48 h. Bacterial translocation was defined as a positive culture of MLNs. Systemic infections were defined as a positive culture of any of the other biological samples, as previously described [54].

### Real-time reverse transcription-polymerase chain reaction (RT-PCR)

Total RNA was extracted from the livers of each group of rats and isolated and purified with TRIzol reagent (Invitrogen, Carlsbad, CA, USA) and NucleoSpin^® ^RNA clean-up kit (Macherey-Nagel, Duren, Germany). The mRNA analysis was carried out by quantitative real-time RT-PCR using LightCycler technology (Roche Diagnostics) for continuous fluorescence detection. The primers for rat leptin receptor isoform b (ob-rb) [55,56], adipoR2 [23] and resistin [57] were used as previously reported and are summarized in Table 5. The reactions were performed using a LightCycler-FastStart DNA Master SYBR Green I Kit (Roche Applied Science) using 50 ng of total RNA extracted from the liver samples. The PCR reaction conditions and the annealing temperature were as previously described [23,55-57]. All samples and standards were amplified in triplicate. The expression level of beta-actin [20] was used as a reference to adjust for an equal amount of sample RNA.

**Table 5:** PCR primers and conditions.

Gene	Primer	Annealing tempertaure°C	Product size (bp)
ob-rb	F: TGCTCGGAACACTGTTAAT	58	171
	R: GAAGAAGAGCAAATATCA		
adipoR2	F: GGATGTGGAAGTCGTGTGTG	56	218
	R: ACCTGGTCAAACGAGACACC		
resistin	F: ACTTCAGCTCCCTACTGCCA	60	273
	R: CTCAGTTCTCAATCAACCGTCC		
beta-actin	F: GTACCCAGGCATTGCTGACA	56	169
	R: TCCTGCTTGCTGATCCACATC		

Ob-rb: the full-length leptin receptor isoform; AdipoR2: adiponectin receptor 2.

### Western blotting

Goat polyclonal IgG antibodies against phospho-signal transducer and activator of Transcription 3 (p-STAT3) (Tyr705), total STAT3 (STAT3), phospho-signal transducer and activator of Transcription 5 (p-STAT5) (Tyr694/Tyr699), total STAT5 (STAT5), phospho-Janus kinase 2 (p-JAK2) (Tyr1007/1008) and total JAK2 (JAK2) were purchased from Santa Cruz Biotechnology (Santa Cruz, CA, USA). Rabbit polyclonal IgG antibodies against phospho-adenosine monophosphate-activated protein kinase-alpha (p-AMPK-alpha) (Thr172), total AMPK-alpha (AMPK-alpha), phospho-extracellular signal-regulated kinase 1/2 (p-ERK1/2) (Thr202/Tyr204), total ERK1/2 (ERK1/2), phospho-c-Jun NH2-terminal kinase (p-JNK) (Thr183/Tyr185), total JNK (JNK), phospho-p38 MAPK (p-P38 MAPK) (Thr180/Tyr182) and total P38 MAPK (P38 MAPK) were obtained from Cell Signaling Technology (Beverly, CA, USA). Rabbit polyclonal IgG antibodies against phospho-peroxisome proliferator-activated receptor -alpha (p-PPAR-alpha) (p-Ser21) and total PPAR-alpha (PPAR-alpha) were purchased from GenScript USA Incorporated (Piscataway, NJ, USA).

Soluble protein was extracted from rat livers using a protein extraction reagent (Pierce, USA) and protein concentration was measured using a bicinchoninic acid assay kit (Pierce, USA). The extracted proteins were resolved by 6-8% sodium dodecyl sulphate-polyacrylamide gel electrophoresis (SDS-PAGE) followed by electrophoretic transfer to nitrocellulose membranes. The membranes were then incubated in 1.0% non-fat dried milk in 50 mM Tris (pH 8.0) followed by incubation overnight with the primary antibodies at 300-500-fold dilution. The bound primary antibody was detected using biotinylated rabbit anti-goat or rabbit anti-rabbit antibody (Zhongshan Goldenbridge Biotechnology, China) and visualized using 3', 3'-diaminobenzidine tetrahydrochloride. Detection was carried out using an electrochemiluminescence kit (Amersham Pharmacia Biotech, UK). Image-Pro Plus software version 6.0 (Media Cybernetics Incorporated, Silver Spring, MD, USA) was used to determine the mean optical density.

### Statistical analyses

Data are mean ± standard deviation. Statistical analyses were done using SPSS software version 13.0 (SPSS Inc., Chicago, IL, USA). A bifactorial ANOVA (followed by post hoc protected least square difference method) were used for statistical comparison. The two fixed factors were treatment (two levels, control treatment/GH treatment) and diet (two levels, control diet/high-fat diet). Weighted kappa values were calculated to determine inter-observer agreement in pathological evaluation. Values of p < 0.05 were considered significant.

## Abbreviations

AMPK-alpha: monophosphate-activated protein kinase-alpha; ERK1/2: extracellular signal-regulated kinase 1/2; GH: growth hormone; GHD: GH deficiency; IGF-1: Insulin-like growth factor 1; IR: insulin resistance; JAK2: Janus kinase 2; JNK: c-Jun NH2-terminal kinase; MDA: malondialdehyde; NAFLD: non-alcoholic fatty liver disease; p38 MAPK: p38 mitogen-activated protein kinase; PPAR-alpha: peroxisome proliferator-activated receptor-alpha; rAAV: recombinant adeno-associated virus; rAAV2/1: recombinant adeno-associated viral vectors pseudotyped with viral capsids from serotype 1; STAT3: signal transducer and activator of transcription 3; STAT5: signal transducer and activator of transcription 5; TNF-alpha: tumour necrosis factor-alpha.

## Competing interests

The authors declare that they have no competing interests.

## Authors' contributions

Guarantor of integrity of entire study YQ and YPT; study concepts and design: YQ and YPT; data acquisition/analysis/interpretation: YQ and YPT, statistical analysis: YQ; obtained funding: YQ and YPT; manuscript drafting or revision for important intellectual content, literature research, manuscript editing, and manuscript final version approval: YQ, and YPT.
